# Systematic Analysis and Biochemical Characterization of the Caffeoyl Shikimate Esterase Gene Family in Poplar

**DOI:** 10.3390/ijms222413366

**Published:** 2021-12-13

**Authors:** Xuechun Wang, Nan Chao, Aijing Zhang, Jiaqi Kang, Xiangning Jiang, Ying Gai

**Affiliations:** 1 College of Biological Sciences and Biotechnology, Beijing Forestry University, Beijing 100083, China; wangxuechun0717@126.com (X.W.); chaonan1989@126.com (N.C.); zhangaijing217@163.com (A.Z.); Kangjiaqi1998@163.com (J.K.); jiangxn@bjfu.edu.cn (X.J.); 2 The Tree and Ornamental Plant Breeding and Biotechnology Laboratory, National Forestry and Grassland Administration, Beijing 100083, China; 3 National Engineering Laboratory for Tree Breeding, Beijing 100083, China; 4 Key Laboratory of Genetics and Breeding in Forest Trees and Ornamental Plants, Ministry of Education, Beijing 100083, China; 5 Jiangsu Key Laboratory of Sericutural Biology and Biotechnology, School of Biotechnology, Jiangsu University of Science and Technology, Zhenjiang 212018, China

**Keywords:** caffeoyl shikimate esterase, enzymatic assay, gene family, lignin, *Populus*

## Abstract

Caffeoyl shikimate esterase (CSE) hydrolyzes caffeoyl shikimate into caffeate and shikimate in the phenylpropanoid pathway. In this study, we performed a systematic analysis of the CSE gene family and investigated the possible roles of *CSE* and *CSE*-like genes in *Populus*. We conducted a genome-wide analysis of the CSE gene family, including functional and phylogenetic analyses of *CSE* and *CSE*-like genes, using the poplar (*Populus trichocarpa*) genome. Eighteen *CSE* and *CSE*-like genes were identified in the *Populus* genome, and five phylogenetic groups were identified from phylogenetic analysis. CSEs in Group Ia, which were proposed as bona fide CSEs, have probably been lost in most monocots except *Oryza sativa*. Primary functional classification showed that *PoptrCSE1* and *PoptrCSE2* had putative function in lignin biosynthesis. In addition, *PoptrCSE2*, along with *PoptrCSE12*, might also respond to stress with a function in cell wall biosynthesis. Enzymatic assay of PoptoCSE1 (*Populus tomentosa*), -2 and -12 showed that PoptoCSE1 and -2 maintained CSE activity. PoptoCSE1 and 2 had similar biochemical properties, tissue expression patterns and subcellular localization. Most of the *PoptrCSE*-like genes are homologs of *AtMAGL* (monoacylglycerol lipase) genes in *Arabidopsis* and may function as MAG lipase in poplar. Our study provides a systematic understanding of this novel gene family and suggests the function of CSE in monolignol biosynthesis in *Populus*.

## 1. Introduction

Lignin is one of the most abundant biomass components in plants and has been extensively studied for decades because of its vital role in the formation of the plant secondary cell wall and wood [1,2,3,4]. The recalcitrance of lignin restricts both forage utilization and processing of biomass for biofuels [5,6]. Therefore, genetic modification strategies targeting key genes involved in monolignol biosynthesis are being developed to obtain plants with less lignin, or with more easily degradable lignin, while maintaining normal biomass [7,8,9,10,11,12].

The monolignol biosynthesis pathway is a branch of the phenylpropanoid pathway and has been continuously revised in recent decades. Eleven enzyme families and twenty-four metabolites have been reported to participate in monolignol biosynthesis [13]. The general phenylpropanoid pathway contains phenylalanine ammonia-lyase (PAL), cinnamate 4-hydroxylase (C4H) and 4-coumarate: CoA ligase (4CL) and provides hydroxycinnamoyl-CoA esters as precursors for a wide range of end products. These hydroxycinnamoyl-CoA esters are further catalyzed into monolignols by a series of enzymes in the specific monolignol biosynthesis pathway [14]. Changing the mass flux in the phenylpropanoid pathway has become a strategy for modifying lignin content [2,5,15,16,17,18]. Recently, dynamic flux modeling for monolignol biosynthesis was also proposed based on systematic understanding of the monolignol biosynthesis pathway [19,20,21,22]. The discovery of caffeoyl shikimate esterase (CSE) as a central enzyme in the lignin biosynthetic pathway promoted a revision of the pathway [18]. *Arabidopsis thaliana cse* mutants have shown that lignin content declines with increasing levels of *p*-hydroxyphenyl units, and the efficiency of the conversion of cellulose to glucose in *cse* mutants is improved after saccharification without pretreatment. CSE together with 4CL directs the flux away from H lignin and toward G and S lignin. Down-regulation of *CSE* in hybrid poplar (*Populus tremula* × *Populus alba*) resulted in up to 25% reduced lignin deposition and increased levels of *p*-hydroxyphenyl units in the lignin polymer and cellulose content [23].

AtCSE (At1g52760), reported as LysoPL2 (Lysophospholipase 2), can be combined with acyl-CoA-binding protein 2 (ACBP2) and lysophosphatidylcholine (lysoPC) to promote the tolerance of *Arabidopsis* to cadmium-induced oxidative stress [24]. Vanholme et al. (2013) reported that caffeoyl shikimate esterase in *Arabidopsis*, AtCSE, had the ability to convert caffeoyl shikimate into caffeate [23]. Both in vitro enzymatic analyses and genetic *cse* mutants confirmed its vital function in monolignol biosynthesis in *Arabidopsis*. Although CSE orthologs were firstly reported based on phylogenetic analysis in several plants such as poplar, it is confused that protein extracts from secondary differentiating xylem of *Populus trichocarpa* showed no detectable CSE activity; similar results were also reported in *Panicum virgatum* and *Oryza sativa* [25]. In a recent study, detectable CSE activity was reported in crude extraction of *P. virgatum* and purified recombinant CSE proteins from *Medicago truncatula* and *Populus deltoides* [26]. The authors also indicated that reaction catalyzed by CSE may not be essential for lignification in all plant species since they found that crude protein extracts from stems of *Brachypodium distachyon* and *Zea mays*, which have no orthologs of the currently characterized *CSE* genes, exhibit only a weak esterase activity with caffeoyl shikimate [26]. In addition, genuine CSE was also characterized in *Pinus* in our previous study [27]. Two bona fide CSEs were characterized in hybrid poplar (*P. tremula* × *P. alba*), and down-regulation of CSE in hybrid poplar resulted in the accumulation of feruloyl shikimate and sinapoyl shikimate, indicating that these compounds also are in vivo CSE substrates [23]. In a recent study, the lignin of *cse1* and *cse2* single mutants in hybrid poplar (*P. tremula* × *P. alba*) by CRISPR-Cas9 had the same levels as the wild type, but both single mutants accumulated caffeoyl shikimate, whereas there was a 35% reduction in lignin in the *cse1 cse2* double mutant [28]. However, Jang et al. (2021) found that the lignin deposition of *cse1* and *cse2* mutants in *P. alba* × *Populus glandulosa* by CRISPR-Cas9 was reduced by 29.1%, although *cse1* and *cse2* were indistinguishable from the wild type in morphology and showed no significant difference in growth [29].

It is necessary to consider all key genes and their homologs involved in monolignol biosynthesis when establishing a dynamic flux model based on enzymatic activity since both the functional redundant and complementation homologs would affect the flux model [19,25]. Therefore, using the poplar (*P. trichocarpa*) genome, which has complete genome information and annotation, we performed a genome-wide analysis of the CSE gene family. Based on the analysis of promoter and expression profiles, we propose the functions of these genes. Biochemical characterization of the putative bona fide CSE homologs from *Populus tomentosa* was also performed in vitro to confirm their catalytic function. Our study provides a further understanding of this novel gene family and new target genes for modifying the phenylpropanoid pathway.

## 2. Results

### 2.1. Organization and Distribution of the CSE Gene Family

**Figure 1:**
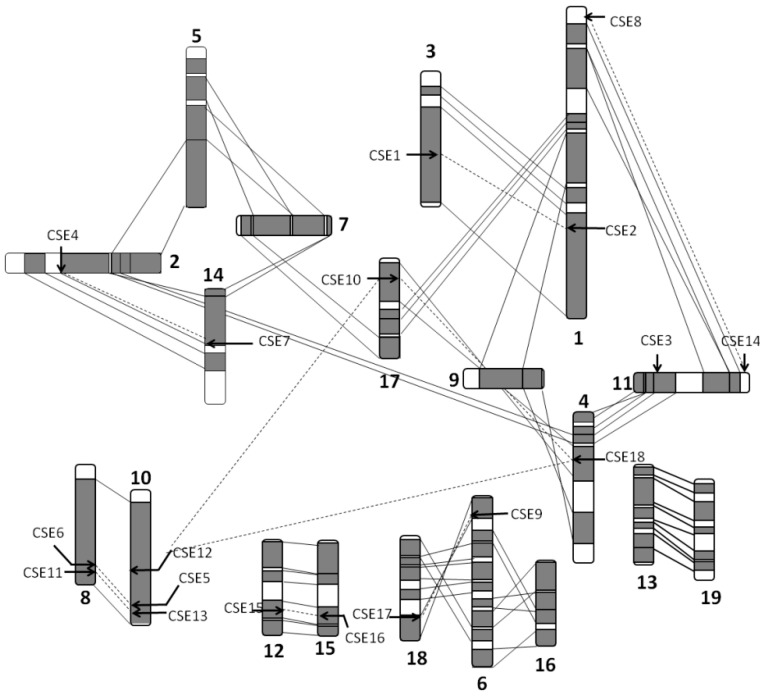
Distribution of *CSE* and *CSE*-like genes on *Populus* chromosomes. Regions that are assumed to correspond to homologous genome blocks are shaded gray and connected by lines. Schematic view of chromosome reorganization from the most recent whole-genome duplication in *Populus* was adapted from Tuskan et al. (2006), based on duplication coordinates from the genome assembly version 3.0. The position of genes is indicated by an arrowhead, and positional paralogs relative to the duplicated blocks are connected by dashed lines. The numbers of 1–18 are chromosome numbers.

We found that the number of CSE gene family members differed among the examined species, and the CSE gene family may have experienced duplication or loss. Eighteen CSE homologs were identified in poplar. These *PoptrCSE* or *PoptrCSE*-like genes are distributed on 13 chromosomes (Figure 1), indicating that block duplication resulting from ‘Salicoid’ genome duplication may play a role in the formation of the CSE family [30]. Four chromosomes, including chromosomes I, VIII, X and XI, contain two or three *PoptrCSE* genes, most of which are located in homologous duplicated blocks and form ten duplicated pairs. *PoptrCSE*3 is located on a duplicated segment on chromosome XI without a matching duplicate. It is possible that its duplicate was lost during evolution. Ks analysis of the duplicated *CSE* gene pairs showed that 7 of the 10 *CSE* gene pairs experienced a recent duplication event, with Ks values between 0.2 and 0.4 (Supplementary Table S1).

**Figure 2:**
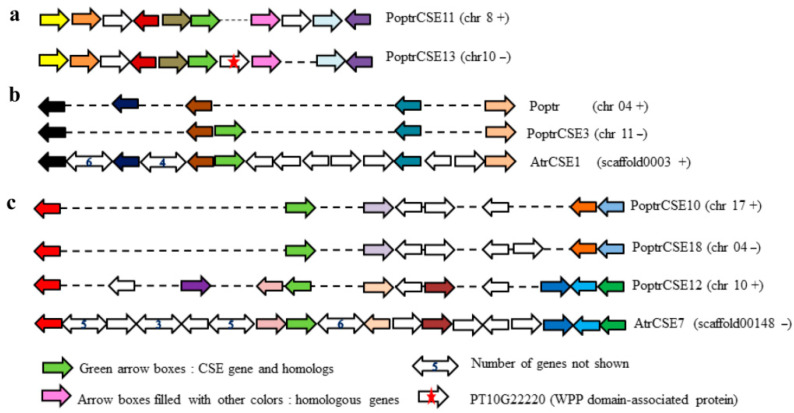
Collinear analysis of homologous segments between *Populus* and *Amborella*. The collinear analysis of (**a**) *PoptrCSE11* and *PoptrCSE13,* (**b**) *PoptrCSE3* and *AtrCSE1* and (**c**) *AtrCSE7*, *PoptrCSE10, PoptrCSE12* and *PoptrCSE18.* Green arrow boxes present the *CSEs* and *CSE* homologs; arrow boxes filled with other colors indicate other homologous genes (orthologs or paralogs). The symbols of *CSE* genes and the chromosomes on which they are located are marked on the right. In particular, Poptr (chr 04 +) means no *CSE* homologs were found on this homologous segment (positive strand) on *Populus* chromosome 04.

The CSE gene family was also screened from a number of plant species, including mosses (*Physcomitrella patens*), ferns (*Selaginella moellendorffii*), gymnosperms (*Picea sitchensis*) and angiosperms, in the present study. Eighteen *PoptrCSE* and *PoptrCSE*-like genes were identified in *P. trichocarpa*. These *PoptrCSE* genes were distributed on 13 chromosomes with homologous pairs, except for *PoptrCSE3*, which was also located in a duplicate block. Inter-species collinear analysis revealed that *PoptrCSE3* is orthologous to the *AtrCSE1* gene in *Amborella,* which is the most primitive branch of angiosperms, while we could not find its positional paralog relative to the duplicated blocks in *Populus* (Figure 2b). In addition, based on the aligned gene strings of a set of homologous segments with *PoptrCSE11* and *PoptrCSE13*, and the fact that *PoptrCSE13* only has a 361 bp upstream sequence, we propose that it is an insertion of a new gene (PT10G22220) into the promoter region of *PoptrCSE13* that leads to this change in the promoter region (Figure 2a). The triangular connection among *PoptrCSE10*, *-12* and *-18* with a high Ks value may be the result of the ancient ‘Salicoid’ genome duplication and following fission and fusion of chromosomes [30,31,32]. We can still observe the collinear relationships among *AtrCSE7*, *PoptrCSE12* and *PoptrCSE18* as well as the loss of genes in gene strings on the homologous segments (Figure 2c). The duplication block containing *PoptrCSE10* is homologous to that with *PoptrCSE18*, indicating that the duplication event occurred between *PoptrCSE10* and *PoptrCSE18*.

### 2.2. Promoter Sequence Analysis

All of the upstream 1500 bp sequences of *PoptrCSE* and *PoptrCSE*-like genes were obtained, except for *PoptrCSE13*, which only has 361 bp to its closest gene. Responsive elements of the six types of hormones were found in different *PoptrCSE* genes, especially *PoptrCSE17*, which had elements responding to the six hormones (Table 1) [33,34,35,36,37,38]. These hormones are important for plant development and defense. AC-rich elements corresponding to the MYB transcription factor-binding motif are necessary for vascular expression [39,40]. *PoptrCSE1*, with the highest similarity to *AtCSE1*, had both the AC-I and AC-II elements in its promoter region. *PoptrCSE2* and *PoptrCSE12* had the AC-I element, and *PoptrCSE5* and -*6* had the AC-II element. In addition, motifs binding to different MYBs were found in many *PoptrCSE* genes. Among these *PoptrCSE* genes, *PoptrCSE18* had two MYB-binding motifs, MBS and MBSI, involved in drought response and flavonoid biosynthetic gene regulation, respectively. Some members of the MYB family have been reported as lignin-specific transcription factors [41,42,43,44]. In addition, we identified a secondary wall NAC binding element (SNBE) and a secondary wall MYB responsive element (SMRE) in the promoter regions of *PoptrCSE* and *PoptrCSE*-like genes, except for *PoptrCSE13* (Supplementary Table S2). Both SNBE and SMRE were reported to be involved in secondary cell wall biosynthesis [45,46]. Furthermore, *cis*-elements involved in the stress response were revealed and were widely detected in the *PoptrCSE2* and *PoptrCSE12* promoter regions.

### 2.3. Alignment and Phylogenetic Analysis of CSE and CSE-like Genes

**Figure 3:**
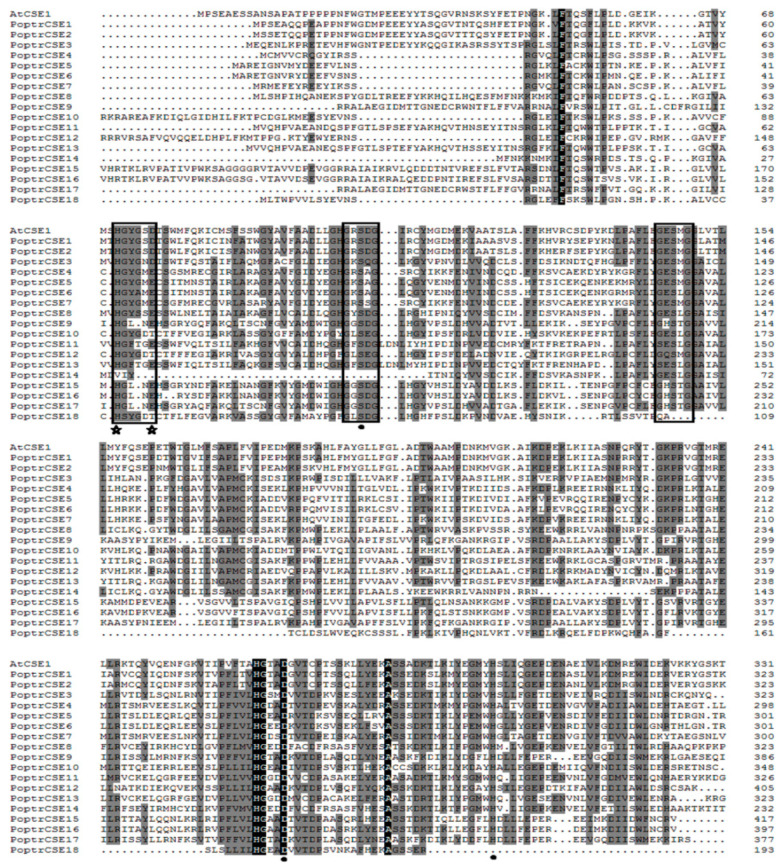
Alignment of eighteen putative PoptrCSE sequences with AtCSE1 (At1g52760). Conserved and functional motifs are indicated in black boxes, including an acyltransferase motif (HX_4_D) and two lipase/hydrolase motifs (GXSXG). Key amino acids involved in enzymatic activity are also marked with black stars (acyltransferase activity) or circles (catalytic triad).

Detailed analyses of putative protein sequences of PoptrCSE and AtCSE1 were also performed (Figure 3). The acyltransferase (HX_4_D) and two hydrolase (GXSXG) motifs were identified in these putative proteins [24,47]. Two GXSXG motifs were highly conserved in all PoptrCSE proteins. HX_4_D, which was reported as a key motif for acyltransferase activity for monoacylglycerol acyltransferase (MGAT) in peanuts, was less conserved [47]. While aspartate and glutamate residues in the sixth position were present in most PoptrCSE proteins, this position was substituted by threonine in PoptrCSE10, -12 and -18. PoptrCSE1, -2 and -3 maintained this position, as with AtCSE1. The catalytic triad (S, D and H), which was reported to be responsible for lipase/esterase activity, was identified as highly conserved in these proteins (black circles) [48]. Another two proteins, PoptrCSE14 and PoptrCSE18, were motif deficient. PoptrCSE14 only had the first hydrolase motif without the other two motifs, and PoptrCSE18 lacked the first hydrolase motif and even appeared to have an incomplete catalytic triad because of its shorter peptide sequence. The above variations indicate a functional change or deficiency in these two proteins.

**Figure 4:**
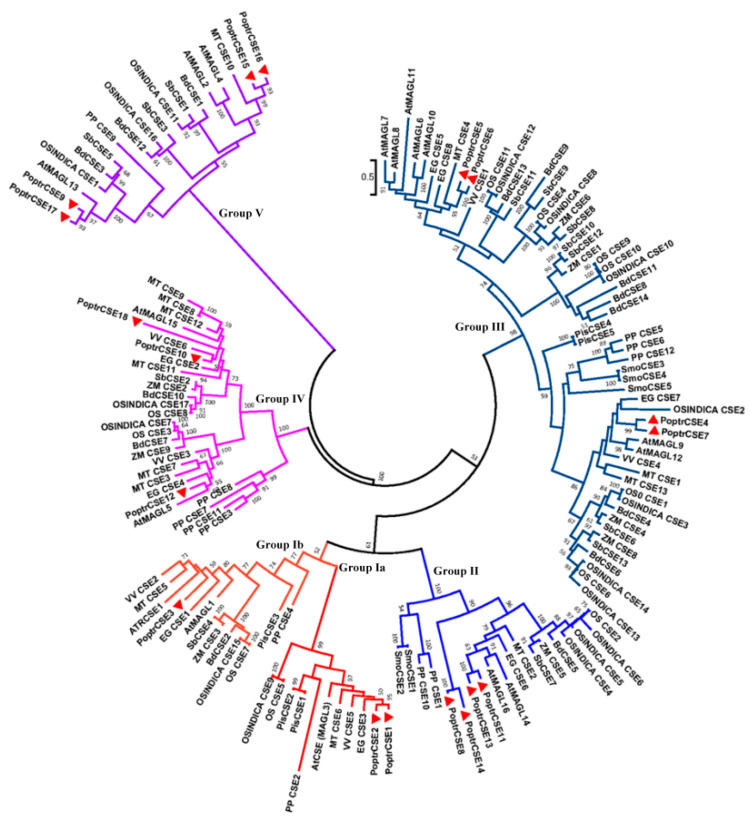
Phylogenetic tree of screened CSE and CSE-like protein sequences from *Populus* and other species. Eighteen PoptrCSE or PoptrCSE-like proteins are marked with red triangles. All 153 CSE and CSE-like proteins including AtMAGLs are listed in Supplementary Table S5.

Phylogenetic analysis was conducted on a total of 153 putative CSE and CSE-like proteins including AtMAGLs (monoacylglycerol lipase) from 18 species. The hydrolase (GXSXG) motifs in these putative proteins were highly conserved, corresponding to the lipase/esterase superfamily, whereas the HX_4_D motifs experienced divergence, especially in the D position, which can be used to reveal the evolutionary relationships between these CSEs. Based on the evolution of the acyltransferase motif and the maximum likelihood (ML) bootstrap phylogenetic tree (Figure 4), all of these putative CSE or CSE-like proteins were divided into five groups. It is noteworthy that most of the CSE or CSE-like proteins in Group I have the classical acyltransferase motif with HX_4_D. Further analysis indicated that this group could be divided into two subsets. Herein, Group Ia is classified as a bona fide CSE group based on the rules described by Raes et al. (2003) [49]. Putative proteins in this subgroup have high sequence identity with AtCSE1, which was identified as a bona fide CSE in the functional analyses [18]. PoptrCSE1 and -2, bona fide PoptrCSEs, were grouped with AtCSE1 in Group Ia and were likely involved in lignin biosynthesis with CSE activity. It is interesting that bona fide CSEs, except two proteins from *Oryza sativa* (OsCSE5 and OSINDICA_CSE7), are found primarily in dicots. CSEs in Group Ib are relatively more closely related to bona fide CSEs. Almost all of the putative proteins in Group II and Group III have HX_4_E instead of HX_4_D. Group IV refers to CSEs with HX_4_T/S. The last group (Group V) contains PoptrCSE15, -16, -17 and -9 and mainly has HX_4_H instead of the acyltransferase motif. In addition, we conducted a separate phylogenetic analysis of PoptrCSE and PoptrCSE-like proteins with AtMAGLs. The phylogenetic analysis also showed that these PoptrCSE-like proteins were clustered with AtMAGLs (Figure S2). PoptrCSE3 (Group Ib) showed a close relationship with AtMAGL1 which has MAG lipase activities and lysophosphatidylcholine (LPC) and/or lysophosphatidylethanolamine (LPE) hydrolase activities. The catalytic residues of putative CSEs are presented in Supplementary Table S3, grouped according to the phylogenetic tree.

### 2.4. Expression Analysis of CSE Genes

**Figure 5:**
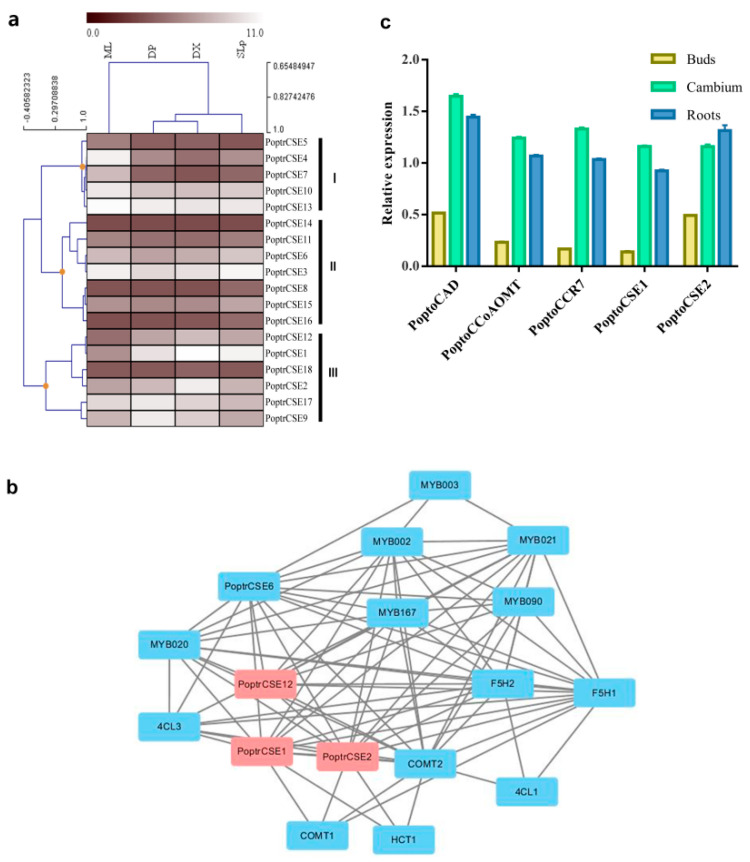
Expression profile and co-expression network of *CSE* and *CSE*-like genes in poplar. (**a**) Expression profile of *PoptrCSE* and *PoptrCSE*-like genes in various *Populus* tissues. Cluster analysis associated with the expression profile is also shown in this figure. Transcript levels are indicated by shading from dark red to white, as shown in the legend. Tissues or specific parts of plants are indicated with the respective abbreviations: DP, developing phloem; DX, developing xylem; ML, mature leaf; SLp, shoot and leaf primordium. The support information including the log2-transformed signal intensity and statistical test is available in Supplementary Table S6. (**b**) Co-expression network of *PoptrCSE* and *PoptrCSE*-like genes with identified genes involved in lignin biosynthesis. *PoptrCSE1*, *-2* and *-12* are marked with red boxes. (**c**) Similar expression pattern of *PoptoCSE1* and *-2*, *PoptoCAD*, *PoptoCCR7* and *PoptoCCoAOMT* using semi-qRT-PCR.

From the results of the hierarchical cluster analysis based on expression levels in different tissues or organs, three expression clusters were found (Figure 5a). *PoptrCSE4*, *-7* and *-10* showed obvious expression preferences in mature leaves and clustered with *PoptrCSE5* and *-13* in cluster I, with a relatively higher expression abundance in mature leaves (ML). *PoptrCSE* and *PoptrCSE*-like genes in cluster II had relatively high expression levels in the shoot and leaf primordium (SLp), with no obvious preferences in all detected tissues and organs. *PoptrCSE1*, *-2*, *-9*, *-12* and *-17*, constituting cluster III, showed expression preferences in developing phloem and xylem, which are involved in wood formation. The co-expression network showed that *PoptrCSE1* and *-2* had a significant correlation with genes involved in lignin biosynthesis, which can be used as evidence to explore the putative function of these *PoptrCSE*s (Figure 5b).

### 2.5. Characteristics of PoptoCSE1 and PoptoCSE2

**Figure 6:**
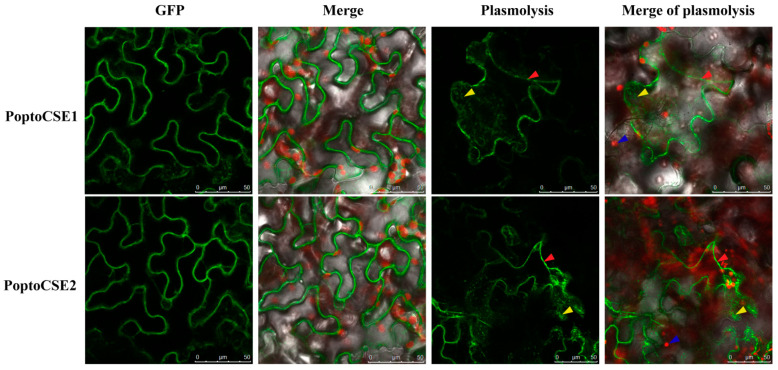
Subcellular localization of PoptoCSE1 and PoptoCSE2 in transformed tobacco leaves. The red arrow, yellow arrow and blue arrow indicate the plasma membrane, ER and chloroplast, respectively.

We further cloned and obtained the corresponding *PoptoCSE1*, *-2* and *-12* from *P. tomentosa*. *PoptoCSE1*, *-2* and *-12* have high sequence identity (>98%) with the corresponding *CSE*s in *P. trichocarpa*. Semi-qRT-PCR (semi-quantitative reverse transcription polymerase chain reaction) showed that *PoptoCSE1* and *PoptoCSE2* had a similar expression pattern to other monolignol biosynthesis-related genes such as *PoptoCAD*, *PoptoCCR7* and *PoptoCCoAOMT* (caffeoyl-CoA 3-O-methyltransferas) (Figure 5c). Both PoptoCSE1 and PoptoCSE2 with fused GFP (green fluorescent protein) were observed in the plasma membrane and ER, and the signal became clearer after plasmolysis (Figure 6). This result corresponds with the subcellular localization of AtCSE (At1g52760), which was reported as LysoPL2 (Lysophospholipase 2) [24].

### 2.6. Enzymatic Assay of PoptoCSE1, -2 and -12 of P. tomentosa

**Figure 7:**
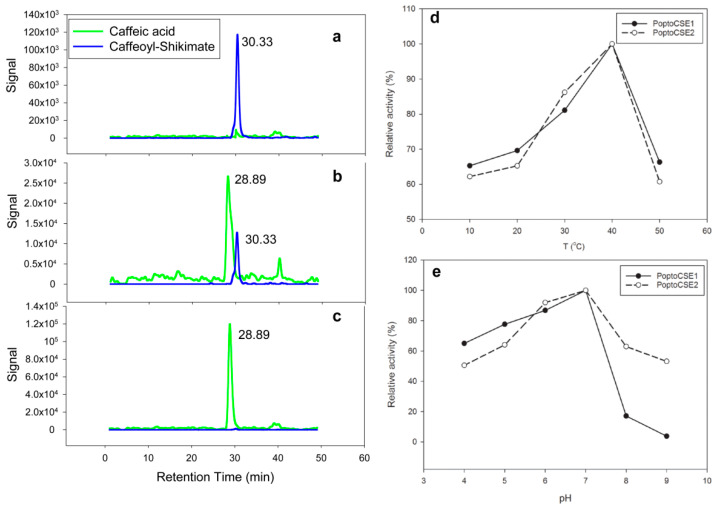
Enzymatic assay of PoptoCSE1 and PoptoCSE2. (**a**–**c**) The decrease in caffeoyl shikimate and the increase in caffeic acid along with the reaction process catalyzed by PoptoCSE1 with different reaction times: 5 s (**a**), 80 s (**b**) and 120 s (**c**). (**d**,**e**) The temperature (**d**) and pH (**e**) profiles for recombinant PoptoCSE1 and PoptoCSE2 activities. The mean value of three replicates was used to calculate the relative activity.

The recombinant proteins PoptoCSE1 and PoptoCSE2 were obtained and purified for enzymatic assay. We detected the CSE activity using HPLC-MS and found that PoptoCSE1 and PoptoCSE2 have detectable activity against caffeoyl shikimate (Figure 7a–c), while PoptoCSE12 showed no activity against caffeoyl shikimate (Figure S1). In addition, we also explored the optimum reaction temperature and pH for PoptoCSE1 and PoptoCSE2 (Figure 7d,e). PoptoCSE1 and PoptoCSE2 have a similar temperature profile and pH profile (except at pH 8.0–9.0) for catalyzing caffeoyl shikimate, and the highest activities for PoptoCSEs were obtained at pH = 7.0 and a temperature of 40 °C. PoptoCSE1 and PoptoCSE2 showed similar biochemical properties against caffeoyl shikimate.

## 3. Discussion

Phylogenetic analysis and comparison of the acyltransferase motif of 153 putative CSEs suggested the existence of 5 CSE and CSE-like groups. PoptrCSE1 and PoptrCSE2 were identified as bona fide CSEs in class I. Additionally, we reported a genuine CSE in the gymnosperm *Larix kaempferi* [27]. The deficiency of CSE in most monocots corresponds to findings of studies in *B. distachyon* and *Z. may* [26]. It is possible that the ancestral CSE appears before the divergence of angiosperms and gymnosperms. The bona fide CSEs (Group Ia) were likely lost in most monocots at the divergence between dicots and monocots. AtCSE was first annotated as LysoPL2 and also identified as a member of the MAGL gene family [24,50]. Here, we further classified these CSE-like proteins into five groups with different amino acid replacements at the last position of the acyltransferase motif. Phylogenetic analysis also showed that these PoptrCSE-like proteins are clustered with AtMAGLs (Figure 4 and Figure S2).

Based on the above analysis in the present study, we primarily proposed functions of *PoptrCSE* and *PoptrCSE*-like genes. *PoptrCSE1*, *-2* and *-12* are expressed mainly in developing xylem (cluster III). Co-expression analysis showed that *PoptrCSE1*, *-2* and *-12* had a significant correlation with genes involved in lignin biosynthesis. The *cis*-elements AC-I, AC-II, SMREs and SNBEs are involved in regulating secondary cell wall biosynthesis [41,46]. It has been reported that MYB58 or MYB63 can bind the AC element, the conserved promoter sequence in most monolignol biosynthetic genes, to directly activate the expression of these genes in *A. thaliana* [51]. *PoptrCSE1*, *-2* and *-12* all contain the AC element (AC-I or AC-II) in the promoter regions. SMREs, additional *cis*-elements along with to the AC elements and *Arabidopsis* MYB46/83, are master switches of the entire secondary wall biosynthetic program and are able to bind to SMREs [52]. Secondary wall NACs (SWNs), as master transcriptional switches activating secondary wall biosynthesis, were reported to bind to SNREs. The SNBE sites binding with SWNs were proposed to be necessary for a combinatorial activation of the secondary wall biosynthesis genes with other transcription factors [53]. In our promoter analysis, SMREs and SNREs were found to widely distribute in most *CSE* and *CSE*-like gene promoter regions, and *PoptrCSE1* and *-2* contained the most SMREs and SNBEs (Table 1). *PoptrCSE1* and *-2* have high sequence identity with *AtCSE1*, both of which have many *cis*-elements involved in secondary wall biosynthesis. The promoter analysis revealed that *PoptrCSE2* and *-12* had many *cis*-elements involved in stress response that differ from *PoptrCSE1*. This indicates that *PoptrCSE1*, -*2* and *-12* are likely involved in wood formation, but *PoptrCSE2* and *PoptrCSE12* might respond mainly to stress. This functional divergence has been reported in other key genes involved in lignin biosynthesis [54,55,56].

In addition, PoptrCSE3, belonging to phylogenetic Group Ib, showed a close relationship with AtMAGL1 which was reported to harbor MAG lipase activities and LPC and/or LPE hydrolase activities. PoptrCSE3 and AtMAGL1 have both acyltransferase (HX_4_D) and hydrolase (GXSXG) motifs and are proposed to have acyltransferase activity. PoptrCSE6, -7, -9, -14, -15, -16 and -17 from phylogenetic groups III and V all have *cis*-elements involved in response to hormones. Previous studies have revealed the biofunction of AtCSE and its function in phospholipid metabolism [24,47]. PoptrCSE9, -15, -16 and -17 clustered with AtMAGL2 and -4 which were reported to have MAG lipase activities and LPC and/or LPE hydrolase activities. In addition, expression of AtMAGL2 and -4 was significantly induced by salt, osmotic and cold stresses. The roles of lipases in jasmonate metabolism and phosphatidic acid signaling associated with plant defense have also been reported [57]. Therefore, CSE-like proteins in this class likely function as lipases and are thought to be involved in plant defense in response to salicylic acid or MeJA. Other *PoptrCSE*-like genes also have analogous AtMAGLs and might also have MAG lipase activity.

## 4. Materials and Methods

### 4.1. Plant Materials

Buds, cambial zone tissue and roots of six-year-old *P. tomentosa* 741 were harvested as plant material in this research from Hebei, China. The cambial zone tissue was collected by cutting the bark longitudinally with a knife, then removing a large piece of the bark and using a scalpel to scrape the cambial zone tissue from the outer layer of the xylem and the inner layer of the phloem. Collected samples were immediately frozen in liquid nitrogen and then stored at −80 °C until use. Three biological replicates were performed for each sample.

### 4.2. Genome-Wide Identification of CSE Gene Family Members

To identify the CSE sequences in *Populus*, we first performed a BlastP search in the NCBI database using AtCSE1 as a query [58]. Sequences from selected species with high scores, query cover > 90%, identity > 50% and a low E-value (1  ^−100^) were collected to build an HMM model. An HMM search was used to find more sequences with the same features using the hidden Markov model, avoiding missing sequences based on alignment. The results of the HMM search were further screened based on scores and E-values (<1  ^−5^) of both the domain and full sequence. Finally, the screening sequences were submitted to the Superfamily 1.75 database (https://supfam.org/SUPERFAMILY/) (access date: 9 December 2021) to further determine their superfamily and family in the SCOP database. Details are available in Supplementary Table S4. The CSE sequences used for phylogenetic analysis (Supplementary Table S5) were screened from various sources based on the platform PLAZA 3.0 (http://bioinformatics.psb.ugent.be/plaza/) (access date: 8 January 2021). CSE sequences from the gymnosperm *P. sitchensis* and the fern *S. moellendorffii* were obtained using BlastP in the NCBI database. The rules proposed by Raes et al. (2003) to distinguish bona fide genes and ‘likes’ were used to determine bona fide *CSE* and *CSE*-like genes [49].

### 4.3. Organization and Distribution of CSE Genes and Homologs on Populus Chromosomes

The identified CSE sequences in *Populus* were submitted to GSDS 2.0 server (http://gsds.gao-lab.org/) (access date: 7 December 2021) to visualize the intron–exon organization patterns of these genes. Then, CSE and CSE homologs were located on their respective chromosomes in specific duplicated blocks based on the *Populus* genome. Duplicated blocks were determined using WGDotplot in the PLAZA platform. A schematic diagram was constructed to describe the organization and distribution of CSE and its homologs on *Populus* chromosomes.

### 4.4. Promoter Analysis of PoptrCSE Genes

Promoter sequences of *PoptrCSE* genes were extracted using the PLAZA platform with the parameter upstream 1500 bp. Promoter analysis was performed by querying all *CSE* genes for the upstream or up to the closest gene using the PlantCARE database (http://bioinformatics.psb.ugent.be/webtools/plantcare/html/) (access date: 30 October 2021) with a selected matrix score of ≥5 for all elements. SNBE and SMRE were detected based on the motif sequences reported by Zhong et al. (2012) [52]. Details are presented in Supplementary Table S2.

### 4.5. CSE Sequence Alignment and Phylogenetic Analysis

Both DNAman 8.0 (Lynnon BioSoft) and CLUSTAL W software assembled in MEGA 5.05 software were used to align candidate PoptrCSEs and bona fide CSEs using default parameters [59]. A phylogenetic tree was obtained using Mega 5.05 with the maximum likelihood method, the JTT substitution model and a model with G + I rates among sites. The reliability of internal branches was assessed by bootstrapping using 1000 bootstrap replicates and marked above nodes only if greater than 50. The putative CSE protein sequences used are listed in Supplementary Table S5.

### 4.6. RNA Extraction and cDNA Synthesis

Total RNA from different tissues was extracted using Plant RN38 Kit (Aidlab, Beijing, China) according to the manual. The quality of RNA was evaluated by both Nanodrop one (Thermo Scientific, Waltham, MA, USA) and electrophoresis. cDNA was synthesized with the PC54-TRUEscript RT kit (Aidlab, Beijing, China) according to the manufacturer’s protocol.

### 4.7. CSE Expression Profile in P. trichocarpa and P. tomentosa

Gene expression profiling for various tissues including DP, DX, ML and SLp in *P. trichocarpa* was performed using the GEO database with the accession number GSE30507. Transcriptome datasets of these samples were re-analyzed by submitting them to Pop’s Pipes: Poplar Gene Expression Pipelines (http://sys.bio.mtu.edu/) (access date: 7 December 2021). In addition, the RNA-seq dataset GSE78953 was also used for confirming *CSE* gene expression, and co-expression profiling based on the Pearson correlation coefficient with genes involved in lignin biosynthesis was also performed for exploring the putative function of *PoptrCSE* and *PoptrCSE*-like genes. GSE78953 contains the transcriptome information of xylem tissue of transgenic and wild-type *P.trichocarpa*. MultiExperiment Viewer (MeV) version 4.9 and Cytoscape 3.4 were used to visualize the *CSE* expression profile or co-expression network. The detailed information is available in Supplementary Table S6. Semi-qRT-PCR was performed for *PoptoCSE1*, *PoptoCSE2* and several other monolignol biosynthesis genes in *P. tomentosa*. Actin was used as a reference gene.

### 4.8. Subcellular Localization of PoptoCSE

The methods used for the subcellular localization of PoptoCSE and plasmolysis were in accordance with our previous study [27]. *CSE*s (*PoptoCSE1*, *-2* and *-12*) were cloned from the cambial zone tissue in *P. tomentosa* based on the sequence information from *P. trichocarpa*. The primer pairs and PCR procedure are listed in Supplementary Table S7. The pBI121 vector with PoptoCSE1 and PoptoCSE2 and GFP fusion expression was constructed by restriction enzymes and then ligation with T4 ligase (Takara, Dalian, China). The recombinant plasmids pBI121-35S-PoptoCSE1 and pBI121-35S-PoptoCSE2 were transferred into *Agrobacterium tumefaciens* strain GV3101, which was transferred into tobacco leaves via *Agrobacterium-mediated* transient transformation [60]. The tobacco leaves were cut into squares of 5 mm × 5 mm, and GFP fluorescence in leaves was observed using a Leica TCS SP8 confocal microscope (Leica Microsystems, Wetzlar, Germany).

### 4.9. Purification of Recombinant CSE and HCT

HCT can catalyze caffeoyl-CoA and shikimic acid to produce caffeoyl shikimate which is the substrate of CSEs. To obtain caffeoyl shikimate, we cloned *HCT* from the cambial zone tissue in *P. tomentosa*. The CSEs and HCT6 were constructed with pET28a (Novagen) and then transformed into *E. coli* BL21 (DE3). The recombinant proteins tagged with His were purified using Ni-NTA agarose (Qiagen). After washing the column using lysis buffer with gradient concentrations of imidazole, the His-tagged PoptoCSEs and PoptoHCT were eluted with 100 mM imidazole in lysis buffer [27]. The eluted proteins were detected by SDS-PAGE and stained by Coomassie Brilliant Blue R250. The concentration of the purified protein was determined using Bradford Protein Assay Kit (Sangon Biotech, Shanghai, China).

### 4.10. Enzymatic Assay of Recombinant PoptoCSEs

We obtained caffeoyl shikimate using enzymatic reaction catalyzed by purified recombinant PoptoHCT6 with caffeoyl-CoA and shikimic acid. The reaction was performed according to the methods provided by Escamilla-Treviño et al. (2014) [61]. Caffeoyl-CoA was chemically synthesized and authenticated as reported in our previous study [62]. Purified PoptoCSEs were incubated at 30 °C for 30 min with 100 mM NaPO_4_ buffer (pH 7.5), 500 μM dithiothreitol and 100 μM caffeoyl shikimate in a final volume of 100 μL [18,23,26]. The control reactions were also performed with proteins that had been boiled for 10 min. The reactions (including controls) were terminated by adding 10 μL of glacial acetic acid. CSE activity was monitored by the appearance of caffeic acid. To explore the optimum reaction temperature, the reactions were incubated at various temperatures that ranged from 10 to 50 °C. Multi-pH phosphate buffers ranging from 4 to 9 were adopted for exploring the optimum reaction pH for CSE. Reaction products with 100 ng sinapic acids which were added as an internal standard were further purified using microporous filters, then injected into an HPLC with a reverse-phase C18 column (ZORBAX 300SB-C18, 2.1 × 150 mm, 3.5 μm; Agilent, Santa Clara, CA, USA) and separated in a step gradient using 1‰ formic acid in water as solvent A and acetonitrile as solvent B. The gradient profile was 100% A, decreased to 65% A in 24 min, then to 0% A in 3 min and maintained for 5 min and then increased to 100% A in 3 min and maintained for 20 min. The flow rate was 0.15 mL/min. The substrates and products were identified using an ion trap mass spectrometer (LCQ DECA XP MAX) coupled with an ESI source (Thermo Finnigan). The parameters used for MS analysis were sheath gas (nitrogen) flow rate, 40 arb; aux/sweep gas (nitrogen) flow rate, 10 arb; spray voltage, 4.5 keV; capillary temperature, 320 °C. The MS raw data are available in Supplementary Table S8.

### 4.11. GenBank Accession Number

The Genbank accession numbers of PtoHCT6 and PtoCSE1, -2 and -12 are KT020989, KT020990, KT020997 and KT021003, respectively.

### 4.12. Statistical Analyses

The correlation analysis and ANOVA analysis were performed using SPSS19.0. Significance was marked at *p* < 0.05.

## 5. Conclusions

In this study, we conducted a systematic analysis of the CSE gene family in poplar and studied the possible role of *CSE* and *CSE*-like genes in poplar. PoptrCSE1 and PoptrCSE2 were identified as bona fide CSEs via phylogenetic analysis. Co-expression analysis showed that *PoptrCSE1*, *-2* and -*12* are significantly related to genes involved in lignin biosynthesis. Additionally, most of the *PoptrCSE*-like genes are homologs of the *AtMAGL* gene in *Arabidopsis* and may act as MAG lipase in poplar. We identified two bona fide CSEs, PoptrCSE1 and PoptrCSE2, by enzymatic assays in vitro and found that PoptoCSE1 and PoptoCSE2 had detectable activity on caffeoyl shikimate, and similar tissue expression patterns and subcellular localization. All these results provide more evidence that two bona fide CSEs are indeed widespread and function in monolignol biosynthesis in *Populus*.

## Figures and Tables

**Table 1:** List of motifs found in the promoter regions of *PoptrCSE* genes in *Populus*. PlantCARE (http://bioinformatics.psb.ugent.be/webtools/plantcare/html/) (access date: 30 October 2021) with a selected matrix score of ≥5 for all elements was adopted to obtain the putative motifs. The number of specific motifs in each gene is marked. More detailed information about all detected motifs is available in Supplementary Table S2. SNBE: secondary wall NAC binding element, SMRE: secondary wall MYB responsive element, LTR: low temperature responsive element, HSE: heat stress responsive element.

	Number of SNBEs	Number of SMREs	Salicylic Acid	MeJA	Gibberellin	Auxin	Abscisic Acid	Ethylene	AC-I Element	AC-II Element	LTR	HSE	Anaerobic Induction	Defense and Stress (TC-Rich Repeats)	MYB Involved in Drought Inducibility (MBS)
PoptrCSE1	4	5	-	-	1	-	1	-	2	1	1	-	2	-	-
PoptrCSE2	4	6	-	-	-	-	1	-	1	-	3	3	-	4	-
PoptrCSE3	1	4	1	-	1	1	-	1	-	-	2	2	5	1	-
PoptrCSE4	4	1	-	-	-	-	1	-	-	-	-	-	2	1	1
PoptrCSE5	6	2	-	-	1	1	-	-	-	1	-	-	2	1	2
PoptrCSE6	4	2	2	-	1	1	1	1	-	1	-	2	3	1	2
PoptrCSE7	3	5	2	1	-	-	2	-	-	-	-	4	1	2	1
PoptrCSE8	5	3	-	1	1	-	1	2	-	-	-	-	2	-	1
PoptrCSE9	3	3	1	-	1	1	-	1	-	-	2	2	5	1	-
PoptrCSE10	2	5	1	2	-	1	1	1	-	-	-	-	3	-	-
PoptrCSE11	2	3	-	-	-	-	1	-	-	-	-	-	5	2	1
PoptrCSE12	5	2	1	1	-	-	1	1	1	-	-	3	1	1	4
PoptrCSE13	0	0	-	-	-	-	1	-	-	-	1	1	-	-	-
PoptrCSE14	7	2	1	2	1	1	1	-	-	-	-	3	1	2	3
PoptrCSE15	5	4	1	3	-	2	1	-	-	-	1	1	-	1	2
PoptrCSE16	5	4	1	3	-	-	-	-	-	-	-	2	1	1	2
PoptrCSE17	2	2	1	2	2	1	3	1	-	-	1	1	-	3	4
PoptrCSE18	2	4	1	1	1	-	-	-	-	1	3	1	4	2	2

## Data Availability

The data presented in this study are available in the article and Supplementary Materials.

## Supplementary Materials

The following are available online at https://www.mdpi.com/article/10.3390/ijms222413366/s1.

## Abbreviations

4CL	4-coumarate: CoA ligase
C3′H	P-coumarate 3-hydroxylase
C4H	Cinnamate 4-hydroxylase
CAD	Cinnamyl alcohol dehydrogenase
CCoAOMT	Caffeoyl-CoA 3-O-methyltransferas
CCR	Cinnamoyl-CoA reductase
DP	Developing phloem
DX	Developing xylem
ER	Endoplasmic reticulum
F5H	Ferulate 5-hydroxylase
GFP	Green fluorescent protein
HCT	Hydroxylcinnamoyl-CoA shikimate/quinate hydroxycinnamoyl transferase
HSE	Heat stress responsive element
LTR	Low temperature responsive element
LysoPL2	Lysophospholipase 2
MAGL	Monoacylglycerol lipase
MGAT	Monoacylglycerol acyltransferase
ML	Mature leaf
PAL	Phenylalanine ammonia-lyase
Semi-qRT-PCR	Semi-quantitative reverse transcription polymerase chain reaction
SLp	Shoot and leaf primordium
SMRE	Secondary wall MYB responsive element
SNBE	Secondary wall NAC binding element
