# Supplementary material for: Systematic Analysis and Biochemical Characterization of the Caffeoyl Shikimate Esterase Gene Family in Poplar
Source: Int J Mol Sci. 2021 Dec 13;22(24):13366. doi: 10.3390/ijms222413366 (PMC8704367; doi:10.3390/ijms222413366)
Supplement: Supplementary file 1 [file ijms-22-13366-s001.zip › Supplementary Figure.pdf]

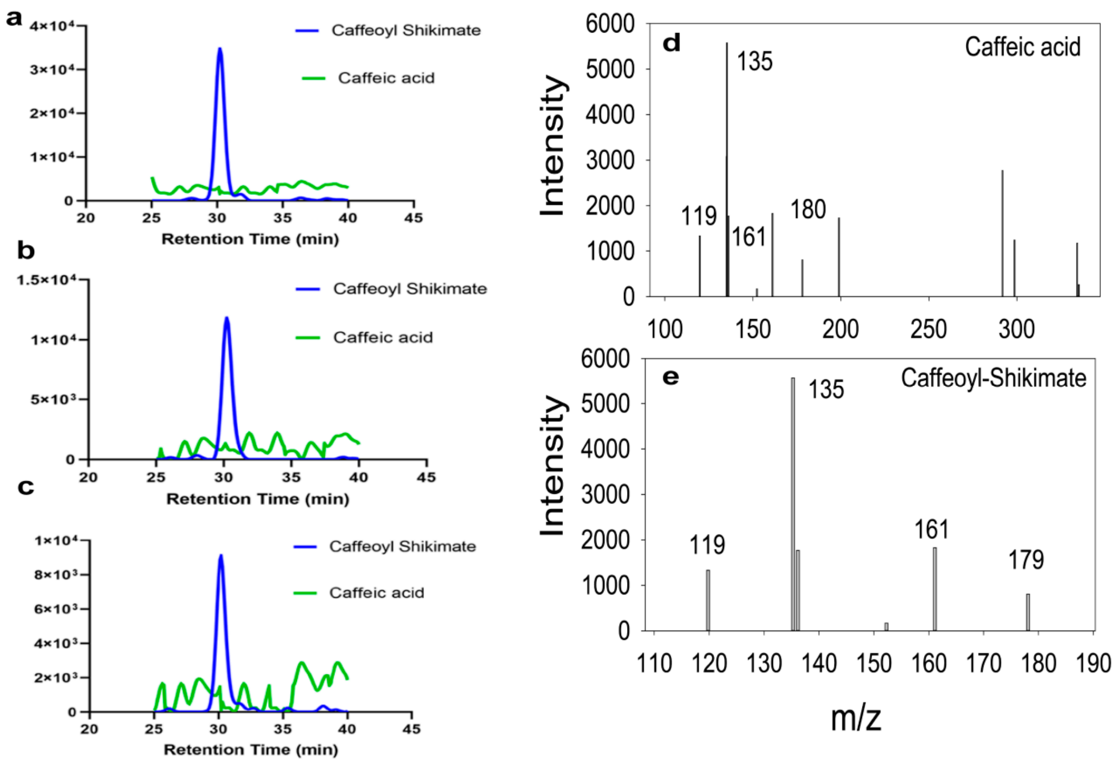

Fig. S1 The decrease of caffeoyl shikimate and the increase of caffeic acid along with the reaction process catalyzed by PoptoCSE12 with different reaction time 0 min (a), 30 min(b) and 60 min (c); HPLC of the caffeic acid (d) and caffeoyl shikimate (e).

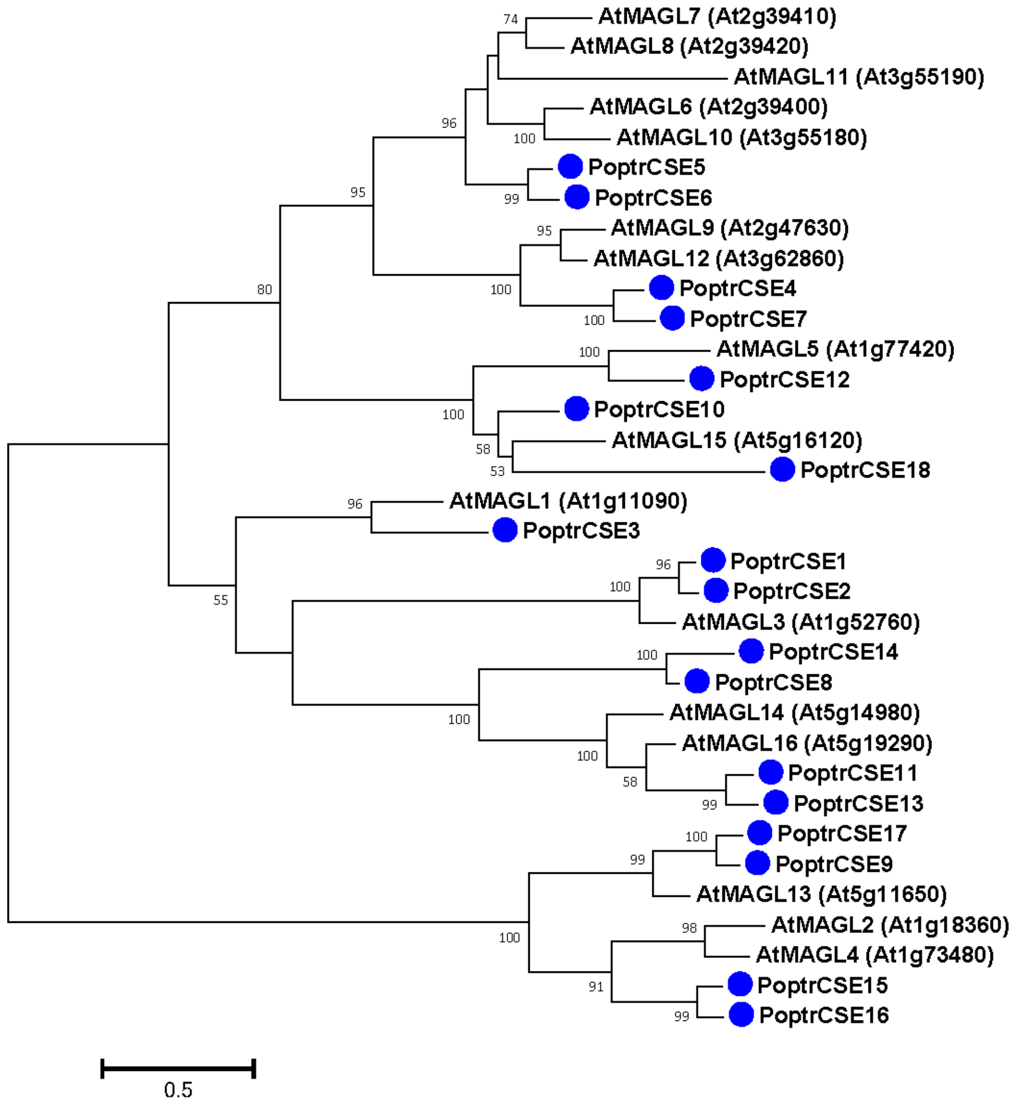

Fig. S2 The phylogenetic analysis of PoptrCSE and PoptrCSE-like proteins with AtMAGLs. Clusters are corresponding with groups in Fig. 4.
